# Peptide Selection of MMP-1 for Electrochemical Sensing with Epitope-Imprinted Poly(TPARA-*co*-EDOT)s

**DOI:** 10.3390/bios12111018

**Published:** 2022-11-15

**Authors:** Mei-Hwa Lee, Cheng-Chih Lin, Piyush Sindhu Sharma, James L. Thomas, Chu-Yun Lin, Zofia Iskierko, Paweł Borowicz, Chien-Yu Lin, Wlodzimierz Kutner, Chien-Hsin Yang, Hung-Yin Lin

**Affiliations:** 1 Department of Materials Science and Engineering, I-Shou University, Kaohsiung 84001, Taiwan; 2 Division of Pulmonary Medicine, Department of Internal Medicine, Armed-Forces Zuoying General Hospital, Kaohsiung 81342, Taiwan; 3 Institute of Physical Chemistry, Polish Academy of Sciences, 01-224 Warsaw, Poland; 4 Department of Physics and Astronomy, University of New Mexico, Albuquerque, NM 87131, USA; 5 Faculty of Mathematics and Natural Sciences, School of Sciences, Institute of Chemical Sciences, Cardinal Stefan Wyszynski University in Warsaw, 01-815 Warsaw, Poland; 6 Department of Chemical and Materials Engineering, National University of Kaohsiung, Kaohsiung 81148, Taiwan

**Keywords:** matrix metalloproteinase-1, epitope imprinting, conductive polymer, electrochemical sensing

## Abstract

Instead of molecularly imprinting a whole protein molecule, imprinting protein epitopes is gaining popularity due to cost and solubility issues. Belonging to the matrix metalloproteinase protein family, MMP-1 is an interstitial collagenase that degrades collagen and may be involved in cell migration, cell proliferation, the pro-inflammatory effect, and cancer progression. Hence, it can serve as a disease protein biomarker and thus be useful in early diagnosis. Herein, epitopes of MMP-1 were identified by screening its crystal structure. To identify possible epitopes for imprinting, MMP-1 was cleaved in silico with trypsin, pepsin at pH = 1.3, and pepsin at pH > 2.0 using Peptide Cutter, generating peptide fragments containing 8 to 12 amino acids. Five criteria were applied to select the peptides most suitable as potential epitopes for MMP-1. The triphenylamine rhodanine-3-acetic acid (TPARA) functional monomer was synthesized to form a stable pre-polymerization complex with a selected template epitope. The complexed functional monomer was then copolymerized with 3,4-ethoxylenedioxythiophene (EDOT) using potentiodynamic electropolymerization onto indium–tin–oxide (ITO) electrodes. The composition of the molecularly imprinted poly(TPARA-*co*-EDOT) (MIP) was optimized by maximizing the film’s electrical conductivity. Cyclic voltammetry was used to determine MMP-1 concentration in the presence of the Fe(CN)_6_^3−^/Fe(CN)_6_^4−^ redox probe actuating the “gate effect.” A calibration curve was constructed and used to determine the usable concentration range and the limit of detection as ca. 0.001 to 10.0 pg/mL and 0.2 fg/mL MMP-1, respectively. Finally, the MMP-1 concentration in the A549 human lung (carcinoma) culture medium was measured, and this determination accuracy was confirmed using an ELISA assay.

## 1. Introduction

Unlike the natural recognizing units of sensors, artificial ones, including molecularly imprinted polymers (MIPs), are used as such units in sensors for determining biomolecular compounds. The development of conductive MIPs has recently improved electrochemical sensing performance [1]. Several monomers have been employed, including pyrrole, phenylenediamine, aniline [2,3], aminothiophenol, aminophenyl boronic acid, aminophenol, thiophene, etc., as well as their copolymers and composites [4]. Instead of imprinting a whole protein molecule [5], protein epitope imprinting is gaining popularity [6], owing to both cost and solubility issues [7,8]. The protein crystal structure and analysis of antibody binding sites, aided by the Protein Data Bank, can be used to identify possible epitopes. Epitopes can be generated by digesting protein in silico with different enzymes using Basic Local Alignment Search Tool (BLAST) software [7,9]. The resulting epitopes can then be categorized according to several criteria, including their location in the protein molecule (exposed or hidden), aggregation properties, and length, optimally between 8 and 12 amino acids. Epitopes satisfying all these criteria are likely to be highly compatible with imprinting.

Matrix metalloproteinase-1 (MMP-1) is an interstitial collagenase that degrades collagen types I, II, and III; its overexpression is implicated in the invasive growth of tumor cells [10]. The biological effects of MMP-1 include migration of keratinocytes, re-epithelialization, cell migration, platelet aggregation, increasing the bioavailability of insulin-like growth factors-1 (IGF-1), cell proliferation, pro-inflammatory effect, and poly(ADP-ribose) polymerase-1 (PARP-1) activation in cancer progression [11]. In pulmonary epithelial cells and skin fibroblasts, environmental factors, e.g., tobacco smoking, increase the expression levels of MMP-1 [12]. A typical MMP consists of several domains, including predomain, propeptide, catalytic, and hemopexin [11]. The propeptide domain, usually composed of ~80 amino acids (AAs), also contains a highly conserved sequence, PRCGVPDV. The catalytic domain consists of ~170 AAs containing conserved three histidine sequences required for zinc chelation. A typical MMP contains a linking peptide, known as a hinge region, of variable length and a hemopexin domain of ~200 AAs [11].

Several assay systems have been developed to diagnose and treat (MMP-expression)-associated diseases [13], including bioassays, zymography assays, immunoassays, fluorimetric assays, radioisotopic-phage-displayed assays, multiple-enzyme/multiple-reagent assays, and activity-based profiling assays. Electrochemical biosensors’ advantages include their low cost and high sensitivity for small molecules [14,15,16], as well as nucleic acids [17,18,19], peptides [3,20,21], and proteins [22,23]. For example, an electrochemical sensor has been fabricated for determining MMP-2 based on the hydrolytic cleavage of specific substrate peptides (PLGVRs). A specially designed ferrocenecetic-acid (Fc)-modified peptide ligand served as the recognition unit of this chemosensor. This peptide was hydrolytically cleaved and then removed from the electrode surface. That decreased the differential pulse voltammetry (DPV) peak current, resulting in a sensor signal that varied linearly with the protein concentration of 1 to 200 ng/mL, with a limit of detection of 0.3 ng/mL [23]. Our previous work demonstrated the optimized molar ratio of functional monomers to templates with the linear dynamic concentration range of 50–500 nM [24].

In the present study, MMP-1 was first screened with BLAST to search for epitope candidates. Using the available crystal structure of MMP-1, these epitope candidates were located in the MMP-1 molecule. The exposed, stable, non-aggregating peptide epitopes selected were next molecularly imprinted in conductive polymers and simultaneously deposited on indium–tin–oxide (ITO) electrodes. The MIP composition was optimized by maximizing the poly(TPARA-co-EDOT) film’s electrical conductivity. Subsequently, the electrode surface morphology was characterized by scanning electron microscopy (SEM). Cyclic voltammetry (CV) was then used to determine MMP-1 in the presence of the Fe(CN)_6_^3−^/Fe(CN)_6_^4−^ redox probe, actuating the “gate effect” [25]. Finally, MMP-1, produced by A549 human lung (carcinoma), was collected in the culture media and then determined with MIP film-coated electrodes.

## 2. Materials and Methods

### Search for the Most Suitable Epitopes of MMP-1 Protein

MMP-1 has the UniProt id of P03956 (MMP-1_HUMAN) and the PDB id of 1fbl. The crystal structure has been determined for 82% of the protein (the remainder being uncrystallizable). The sequence of MMP-1 is shown in Figure S1 in the Supplementary Materials (https://www.uniprot.org/uniprot/P03956.fasta, accessed on 14 December 2022). To identify possible epitopes for imprinting, MMP-1 was cleaved in silico with trypsin, pepsin at pH = 1.3, and pepsin at pH > 2.0 using *Peptide Cutter* (https://web.expasy.org/peptide_cutter/, accessed on 14 December 2022), generating peptide fragments containing 8 to 12 amino acids. Five criteria were applied to select peptides most suitable as potential epitopes for MMP-1.

The first criterion considered was peptide structural stability, represented by the instability index. A protein whose instability index is lower than 40 is predicted to be stable; a value exceeding 40 predicts that the structure is unstable (https://web.expasy.org/protparam/protparam-doc.html, accessed on 14 December 2022).

The second one was aggregation “hot spots.” Aggregating peptides, whose aggregation “hot spots” differed from zero, were considered unsuitable for imprinting.

The third criterion was hydrophobicity, represented by the GRAVY index. Hydrophilic peptides are preferable, as they are more likely to be on the target protein’s surface and provide a uniform environment for chemosensor recognition.

The fourth criterion was selectivity, represented by the E value of the peptide epitope. This criterion distinguishes peptides with the highest possibility of being found only in the MMP-1 protein molecule.

The last criterion involved the cleaved epitopes’ location on the surface of the MMP-1 molecule. All the peptides were sorted according to the above criteria (Table S1 in Supplementary Materials). The most recommended peptide candidates are at the top of this table [25].

## 3. Results and Discussion

Figure S2 in the Supplementary Materials shows the multi-cyclic potentiodynamic curve for simultaneous formation by electropolymerization and deposition on the electrode of the non-imprinted polymer (NIP) and MIP films of various molar ratios of TPARA-to-EDOT and 0–0.5 μg/mL of the MMP-1 peptide epitopes, vis., AQDDIDGIQAI (peptide A, PA). In Figure S2a, the most pronounced anodic and cathodic peaks, at ~0.75 and ~0.70 V vs. Ag/AgCl, respectively, were obtained in the template absence, apparently corresponding to the TPARA redox behavior. Although no pronounced peak was at other potentials, the current increased with the cycle number, implying that the EDOT and TPARA moieties were simultaneously electropolymerized. Thus, a conductive polymer film was deposited on the ITO surface. The peak current increase in the curve for TPARA:EDOT = 1:4 (Figure S2b) with the template concentration increase was less pronounced than that obtained in the absence of the template (Figure S2a). For TPARA:EDOT = 1:1, the peak currents (Figure S2c) with the template presence were higher than those in its absence. These currents did not increase in consecutive cycles. Presumably, the simultaneous electropolymerization and deposition are dominated by the TPARA, not the EDOT moiety. Notably, the peak currents in Figure S2c are much higher than those in Figures S2a,b. As the TPARA:EDOT molar ratio was increased to 4:1 (Figure S2d), peak currents were similar in shape but higher than those for the equimolar ratio (Figure S2c).

**Figure 1:**
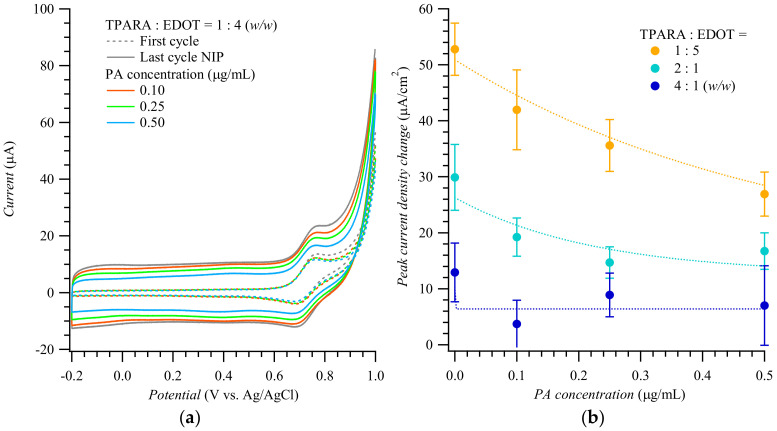
(**a**) Potentiodynamic curves of the 1st and 20th cycle during simultaneous electropolymerization and deposition of PA-templated MIP films for different concentrations of the peptide A template. (**b**) The difference in the peak current of the 20th and 1st polymerization cycle, for different TPARA-to-EDOT ratios, vs. PA template concentration.

Figure 1a shows the 1st and 20th (last) cycle curves of the multi-cyclic potentiodynamic curve for the MIP film deposition displayed in Figure S2. The peak current increased with the cycle number. However, the higher the 20th cycle peak current, the less PA was doped in the MIP. Figure 1b shows differences in peak currents against the PA concentration for the 20th and 1st electropolymerization cycle at various TPARA-to-EDOT molar ratios. This difference was the highest at the 1:4 molar ratio, implying that TPARA incorporation in the TPARA and EDOT copolymer enhances the electrode process. The peak current difference was much smaller for the TPARA-to-EDOT ratio of 1:1, indicating that TPARA and EDOT hinder each other’s electropolymerization. At intermediate TPARA:EDOT ratios, the peak current difference varied monotonically with the TPARA concentration increase. Remarkably, the lower the peak current difference, the higher the template PA concentration for all TPARA-to-EDOT ratios, inferring that PA incorporation inhibits the electropolymerization of TPARA and EDOT. Our previous work demonstrated the interactions between the peptides of C-reactive protein during electropolymerization [3]. The current intensity of the oxidation peak decreased with the increase in PA concentration, revealing that the latter decreases the electropolymerization/electrodeposition rate of polyaniline, perhaps because of the –NH_2_ functional group present on the PA side chains [3].

**Figure 2:**
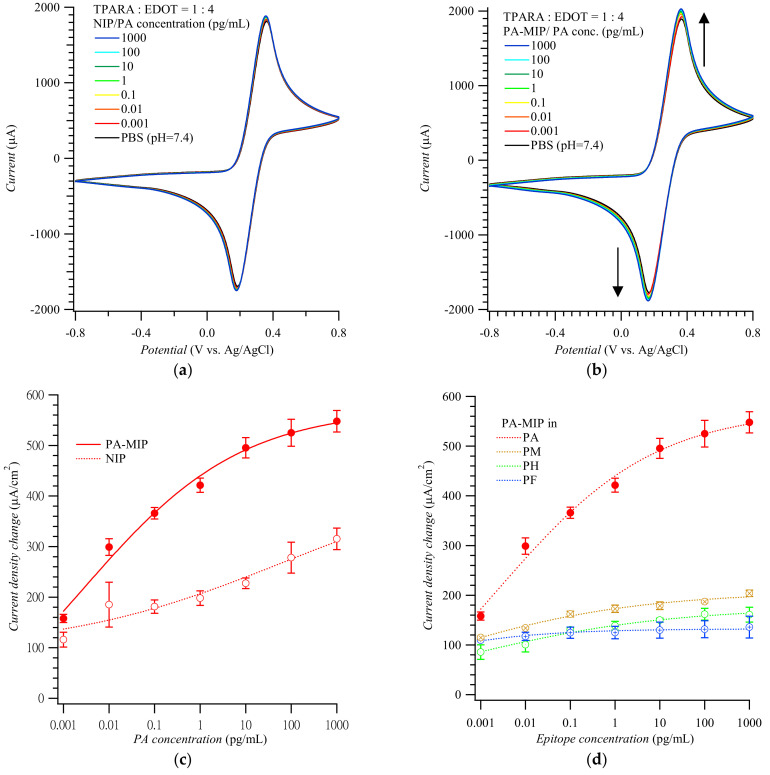
Cyclic voltammograms for target epitopes at different concentrations using (**a**) the NIP- and (**b**) PA-templated poly(TPARA-*co*-EDOT) film-coated electrodes; vertical arrows indicate current changes in consecutive cycles. (**c**) Calibration plots for the PA analyte epitope from (**a**,**b**). (**d**) Interfering effect of other epitopes from MMP-1 at various concentrations. Peak current changes were measured at anodic peak potentials of 0.24 to 0.29 V vs. Ag/AgCl in 125 mM KCl, 5 mM K_4_Fe(CN)_6_, and 5 mM K_3_Fe(CN)_6_. Peptides PA, PH, PM, and PF are AQDDIDGIQAI, HGYPKDIYSS, MIAHDFPGIGHK, and FKGNKYWAVQGQNV, respectively.

**Figure 3:**
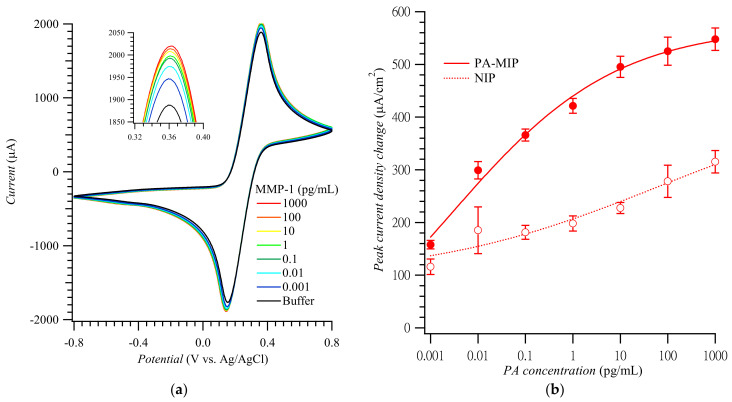
(**a**) CV curves and (**b**) calibration plots of the anodic peak current density change vs. MMP-1 concentration constructed using PA-templated poly(TPARA-*co*-EDOT) film-coated electrodes in 5 mM K_4_Fe(CN)_6_ and 5 mM K_3_Fe(CN)_6_ (adapted with permission from Ref. [21]). The anodic peaks were measured in the range of 0.34 to 0.38 V vs. Ag/AgCl.

Figure 2 shows CV voltammograms for the Fe(CN)_6_^3−^/Fe(CN)_6_^4−^ redox probe used to actuate the “gate effect” [17,18,26] at the NIP (Figure 2a) and MIP (Figure 2b) film-coated electrode in the presence of the PA analyte of different concentrations. CV curves in Figure 2a,b display pairs of cathodic and anodic peaks at ~0.20 and ~0.40 V vs. Ag/AgCl, respectively, representing reversible electro-oxidation and electroreduction of the probe. Apparently, the conductive polymer film amplified the peaks of the redox probe to ~2 mA. Expectedly, the peak current change, which accompanied the electrode transfer from the blank PBS solution to that of the (PA analyte)-containing PBS, was higher for the MIP than for the NIP film-coated electrode. The anodic peak current differences in Figure 3a,b were then replotted in Figure 2c as a function of PA concentration. Differences in the anodic peak currents on the MIP film-coated electrode drastically increased when PA reached 1.0 fg/mL. The MIP film-coated electrode selectivity was also examined (Figure 2d). The CV anodic peak current differences for other MMP-1 epitopes, including PA, PH, PM, and PF, varied from 100 to 200 μA/cm^2^; i.e., they were even lower than those for the PA template on the NIP film-coated electrode.

SEM images of NIP and MIP PA-templated poly(TPARA-*co*-EDOT) film-coated electrodes are displayed in Figure S3 in the Supplementary Materials. From top to bottom, the graphs show films before and after PA template removal from the film, then after PA analyte binding from its 1.0 pg/mL solution for 30 min. The SEM image of the NIP poly(TPARA-*co*-EDOT) film-coated electrode before PA template extraction (Figure S3a) shows granular surface deposits with some raised particles. The average granule size of the MIP PA-templated poly(TPARA-*co*-EDOT) film deposited from 0.5 μg/mL PA (right column in Figure S3) was smaller than that of the NIP film-coated electrode (left column in Figure S3). Results of EDX elemental analyses of the non-templated and PA-templated poly(TPARA-co-EDOT) are summarized in Table 1. The atomic carbon-to-nitrogen-to-oxygen-to-sulfur ratios were ca. 30:1:15:3, indicating that the EDOT-to-TPARA molar ratio was the same as in the stock solution of the 4:1 molar ratio.

Figure S4 shows the C 1s deconvoluted XPS spectra; the C 1s envelope could be decomposed into aromatic C, C-S, C=C-O, and C-O-C components corresponding to the binding energies of 284.7, 285.3, 286.1, and 286.9 eV, respectively. The deconvolution results are listed in Table 2. The aromatic C component decreased, and C-S, C=C-O, and C-O-C components increased after NIP film washing, indicating that the TPARA moiety is its primary unreacted component. On the PA analyte-bound NIP film, the aromatic C component recovered to the level characteristic of that before washing, whereas the C-S and C-O-C EDOT components’ content was decreased. However, the C=C-O component content increased after PA analyte binding, arising from PA’s presence. For pAIPs, both the aromatic C and C-S contents decreased. However, the latter decreased more significantly after washing. Furthermore, both C=C-O and C-O-C components’ contents increased. However, the latter increased more significantly after washing, indicating that TPARA is the primary unreacted component in the MIP (as was the case for the NIP). TPARA and EDOT moieties did not completely react and were deposited at the same molar ratio. On the PA analyte-bound MIP film, the aromatic C component almost recovered to the level characteristic of that before washing, whereas the C-S and C=C-O components’ contents slightly increased, and the C-O-C components’ content significantly decreased.

Finally, the CV peaks for NIP and MIP (Figure S5a,b, respectively), with the PA template extracted, film-coated electrodes in 5 mM K_4_Fe(CN)_6_ and 5 mM K_3_Fe(CN)_6_, 1.0 pg/mL PA, and 1.0 pg/mL MMP-1 in the PBS (pH = 7.4) solution, obeyed the Randles–Ševčik equation of *i*_p_ = 268600 *n*^3/2^ *A D*^1/2^ *c v*^1/2^ at 25 °C, where *i*_p_ is the peak current in amperes, *n* is the number of electrons transferred, *A* is the electrode surface area in cm^2^, *F* is the Faraday constant in C/mol, *D* is the diffusion coefficient in cm^2^/s, *c* is the concentration in mol/cm^3^, *ν* is the potential scan rate in V/s, *R* is the gas constant in J/K mol, and *T* is the temperature in K. The ac impedance measurements for MIP and NIP film-coated electrodes in 5 mM K_4_Fe(CN)_6_ and 5 mM K_3_Fe(CN)_6_, 1.0 pg/mL PA, and in the PBS solution (Figure S3c) indicated that the charge transfer resistance (*R*_ct_) values were ~30, 28, 22, and 22 Ω, respectively. Moreover, the resistances for MIP film-coated electrodes were lower in the 1.0 pg/mL PA analyte solution than in the plain PBS solution. Figure S5d illustrates the reusability of the MIP film-coated electrodes treated with the same washing procedures as those for the template removal described in the Supplementary Materials. The MMP-1 analyte was then bound from its 1.0 pg/mL solution by the MIP film-coated electrode. The MMP-1 analyte could be determined at least five times without the electrode losing its sensing performance and with a standard deviation of less than 7.3%.

Figure 3a shows CV curves for the MMP-1 of different concentrations. These curves’ anodic peak current densities were replotted to construct the MMP-1 calibration plots for the MIP and NIP film-coated electrodes (Figure 3b) [21]. The imprinting factor, calculated as the ratio of slopes of the calibration plots for the MMP-1 at the MIP to that at the NIP film-coated electrodes in the MMP-1 determination range, was ca. 2.6–3.3. The linear dynamic concentration range and the limit of detection were ca. 0.001 to 10.0 pg/mL and 0.2 fg/mL MMP-1, respectively, which are comparable with other works listed in Table S2.

The calibration plot for the MIP film-coated electrode was then employed for MMP-1 determination in the culture medium of A549 cells. The MMP-1 concentration, determined using the MIP films-coated electrodes, was successfully confirmed using ELISA (Table S2). Imaging of A549 cells (Figure S6 in Supplementary Materials) shows optical, DAPI, anti-(MMP-1)-stained, and merged images from the top left to the bottom right. As expected, there was a pronounced expression of MMP-1 within the cells, as seen in the merged image.

## 4. Conclusions

Towards the fabrication of a cost-effective MIP-based chemosensor, we adopted a stepwise approach to finding suitable epitopes of a costly MMP-1 protein biomarker. BLAST software was helpful in the search for the most appropriate MMP-1 peptide epitopes. Then, the chosen MMP-1 epitope was successfully imprinted in the poly(TPARA-*co*-EDOT) film during its simultaneous formation and deposition. For the epitope and whole-MMP-1 protein selective determination, the Fe(CN)_6_^3−^/Fe(CN)_6_^4−^ redox couple was used as the probe in a “gate effect” transduction applied [25,26]. The imprinting factor was ca. 2.6–3.3. The MMP-1 detectability was at a picogram level; thus, the fabricated chemosensor is suitable for MMP-1 determination in real samples of cancerous tumor human cells, as was demonstrated using A549 cells in tissue culture [21].

## Figures and Tables

**Table 1:** Results of EDX elemental analyses of the PA-templated poly(TPARA-*co*-EDOT).

Polymer	Polymer Treatment	Element Content (Atomic %)			
		Carbon	Nitrogen	Oxygen	Sulfur
NIP	BW	61.8	2.1	30.3	5.7
	AW	70.5	1.3	21.9	6.4
	RB	66.2	3.4	25.1	5.4
MIP	BW	70.2	1.5	22.0	6.4
	AW	69.6	1.9	22.3	6.2
	RB	71.2	2.9	20.5	5.4

BW—before washing; AW—after washing; RB—after PA analyte binding from the 1.0 pg/mL PA solution for 30 min.

**Table 2:** Results of XPS elemental analyses of the PA-templated poly(TPARA-*co*-EDOT) using deconvoluted C 1s bands.

Polymer	Polymer Treatment	Element Content (Atomic %)			
		Aromatic C	C-S	C=C-O	C-O-C
NIP	BW	56.94	0.62	23.3	19.14
	AW	52.73	1.17	25.11	20.99
	RB	55.79	0.85	29.38	13.99
pAIP	BW	58.82	9.83	22.8	8.54
	AW	43.97	1.1	28.9	26.03
	RB	61.71	1.61	32.82	3.86

BW—before washing; AW—after washing; RB—after PA analyte binding from the 1.0 pg/mL PA solution for 30 min.

## Data Availability

The authors confirm that the data supporting the findings of this study are available within the article and its Supplementary Materials.

## Supplementary Materials

The following supporting information can be downloaded at: https://www.mdpi.com/article/10.3390/bios12111018/s1, S1. Experimental methods; Table S1. Peptides and their physico-chemical properties resulting from in silico cleavage of the MMP-1 biomarker; Table S2. The comparison of sensing methods for MMP-1; Table S3. MMP-1 determination in the culture medium of A549 cells using the MIP film-coated electrode chemosensors and the ELISA; Scheme S1. A proposed structural formula of the pA-imprinted poly(TPARA-*co*-EDOT). Figure S1. FASTA form of matrix metalloproteinase-1 (MMP-1) biomarker; Figure S2. Potentiodynamic curves for deposition of (a) non-imprinted (NIP) and peptide-imprinted polymer (MIP) films with 0.50 mg/mL PA present during the electropolymerization in a TPARA:EDOT at (b) 1:4 (*wt*/*wt*) (c) 1:1, and (d) 4:1 solution on 0.5 × 0.5 cm^2^ I.T.O. electrodes; Figure S3. S.E.M. images of (a) NIPs, and (b) PA-templated poly(TPARA-*co*-EDOT) film-coated electrodes (a’) before, (b’) after PA template removal with ethanol, and (a” and b”) after PA analyte binding from the 1 pg/mL PA solution for 30 min; Figure S4. The XPS spectra of non-deconvoluted and deconvoluted C 1s bands for the (a) non-imprinted (NIP) and (b) PA-templated poly(TPARA-*co*-EDOT) (PA-MIP) films; Figure S5. The CV peaks for (a) the NIP and (b) MIPs in 5 mM K_4_Fe(CN)_6_ and 5 mM K_3_Fe(CN)_6_, 1.0 pg/mL PA and 1.0 pg/mL MMP-1, in the PBS (pH = 7.4) solution, fitted with the Randles–Ševčik equation. (c) The ac impedance imaginary vs. real component dependence for the MIP and NIP film-coated electrodes in 5 mM K_4_Fe(CN)_6_ and 5 mM K_3_Fe(CN)_6_, in the 1.0 pg/mL pA, PBS (pH = 7.4) solution and a similar solution without PA. (d) The reusability of the PA-templated (pAIPs) poly(TPARA-*co*-EDOT) film-coated electrodes. The electrode was used to determine MMP-1 in its 1.0 ng/mL solution, subsequently rinsed, then reused five times; Figure S6. (a) optical, (b) DAPI staining, (c) immunohistochemistry of MMP-1 proteins, and (d) merged images of A549 lung cancer cells at 400X magnification. References [14,16,24,27,28,29,30,31,32,33,34,35] are cited in the supplementary materials.
