# Supplementary material for: Peptide Selection of MMP-1 for Electrochemical Sensing with Epitope-Imprinted Poly(TPARA-co-EDOT)s
Source: Biosensors (Basel). 2022 Nov 15;12(11):1018. doi: 10.3390/bios12111018 (PMC9688374; doi:10.3390/bios12111018)
Supplement: Supplementary file 1 [file biosensors-12-01018-s001.zip › biosensors-2009931-supplementary.pdf]

biosensors

Peptide Selection of MMP-1 for Electrochemical Sensing with Epitope-imprinted Poly(TPARA-*co*-EDOT)s

Mei-Hwa Lee [1,†], Cheng-Chih Lin [2,†], Piyush Sindhu Sharma [3], James L. Thomas [4], Chu-Yun Lin [5], Zofia Iskierko [3], Paweł Borowicz [3], Chien-Yu Lin [5], Wlodzimierz Kutner [3,5,*], Chien-Hsin Yang [6,*], and Hung-Yin Lin [6,*]

1 Department of Materials Science and Engineering, I-Shou University, Kaohsiung 84001, Taiwan
2 Division of Pulmonary Medicine, Department of Internal Medicine, Armed-Forces Zuoying General Hospital, Kaohsiung 81342, Taiwan
3 Institute of Physical Chemistry, Polish Academy of Sciences, 01-224 Warsaw, Poland
4 Department of Physics and Astronomy, University of New Mexico, Albuquerque, NM 87131, USA
5 Faculty of Mathematics and Natural Sciences, School of Sciences, Institute of Chemical Sciences, Cardinal Stefan Wyszynski University in Warsaw, 01-815 Warsaw, Poland
6 Department of Chemical and Materials Engineering, National University of Kaohsiung, Kaohsiung 81148, Taiwan
* Correspondence: wkutner@ichf.edu.pl (W.K.); yangch@nuk.edu.tw (C.-H.Y.); linhy@caa.columbia.edu (H.-Y.L.)

Citation: Lee, M.-H.; Lin, C.-C.; Sharma, P.S.; Thomas, J.L.; Lin, C.-Y.; Iskierko, Z.; Borowicz, P.; Lin, C.-Y.; Kutner, W.; Yang, C.-H.; et al. Peptide Selection of MMP-1 for Electrochemical Sensing with Epitope-Imprinted Poly(TPARA-*co*-EDOT)s. *Biosensors* **2022**, *12*, 1018. https://doi.org/10.3390/bios12111018

Received: 19 October 2022
Accepted: 10 November 2022
Published: 15 November 2022

Publisher's Note: MDPI stays neutral with regard to jurisdictional claims in published maps and institutional affiliations.

Copyright: © 2022 by the authors. Submitted for possible open access publication under the terms and conditions of the Creative Commons Attribution (CC BY) license (https://creativecommons.org/licenses/by/4.0/).

S1. Experimental

S1.1 Reagents

MMP-1 was in silico cleaved with the trypsin, pepsin (pH = 1.3), and pepsin (pH > 2.0) enzymes from Peptide Cutter (https://web.expasy.org/peptide_cutter/). The MMP-1 peptide epitopes, vis., AQDDIDGIQAI (peptide A, PA), HGYPKDIYSS (peptide H, PH), MIAHDFPGIGHK (peptide M, PM), and FKGNKYWAVQGQNV (peptide F, PF), explored in Section 2.1, were purchased from Yao-Hong Biotechnology Inc. (HPLC grade, New Taipei City, Taiwan). 3,4-Ethoxylenedioxythiophene (EDOT) was from Sigma-Aldrich Co. (St. Louis, MO, USA) and Acros Organics (New Jersey, USA) [1]. Recombinant human MMP-1 protein was purchased from abcam (ab254309). Non-small lung cancer cell lines A549 (BCRC #60074, Food Industry Research and Development Institute, Hsinchu, Taiwan) were cultured in F-12K medium (10% fetal bovine serum, FBS, 1.5 g/L $NaHCO_3$, 20 mM L-glutamine, and 1% penicillin-streptomycin). Deionized (DI) water with a resistivity of 18 MΩ cm (PURELAB, ELGA) was used for preparing the solutions. All chemicals were used as received unless otherwise mentioned.

S1.2 Synthesis of triphenylamine rhodanine-3-acetic acid (TPARA)

Triphenylamine rhodanine-3-acetic acid (TPARA) was synthesized according to the previously reported procedure [2]. Briefly, 4-formaltriphenylamine (150 mg, 0.55 mmol) and rhodanine-3-acetic acid (110 mg, 0.57 mmol) were added to glacial acetic acid (15 mL). Next, this reaction mixture was refluxed for 3 h in the presence of ammonium acetate (100 mg). After cooling to room temperature, it was poured into ice water. Subsequently, the obtained precipitate was filtered out, then washed with DI water. After drying under decreased pressure, the precipitate was recrystallized from ethanol, resulting in red crystals of TPARA (190 mg, yield 75%) [3].

S1.3 Deposition of Epitope-Imprinted poly(TPARA-co-EDOT) Films on Electrodes

Epitope concentrations were either 0 (for non-imprinted polymer film-coated control electrodes) or 0.5% in an ACN solution of 1 mM (total) of TPARA (functional monomer 1, FM1) and EDOT for electropolymerization. Then indium-tin-oxide (ITO) electrodes were immersed in this solution, in the epitope presence or absence, to prepare (epitope template)-molecularly imprinted (MIP) and non-imprinted (NIP) polymer film-coated electrodes, respectively. Subsequently, the resulting conductive polymer film-coated ITO

electrodes were connected to a CHI 400C potentiostat (CH Instruments, Inc., Austin, TX, USA), and the potential was cycled at a 0.1 V/s scan rate, between -0.60 and 0.60 V vs. Ag/AgCl [4]. These electrodes were then immersed in a 10-mL sample of 5 vol% ethanol solution and then rotated at 130 rpm for 5 min on an OSR201-1 orbital shaker (GenePure technology, Taichung, Taiwan). This step was repeated using DI water. Finally, the same washing procedure was applied to remove the MIP-bound epitope for the epitope-imprinted MIP film-coated electrodes' reusability measurements.

S1.4 Characterization of Epitope-Imprinted poly(TPARA-co-EDOT) Conductive Films

The electrochemical reactions at electrodes were controlled and monitored with a CHI 608-1A potentiostat (CH Instruments, Inc., Austin, TX, USA). A solution (10 mL) of 125 mM KCl, 5 mM $K_4Fe(CN)_6$, and 5 mM $K_3Fe(CN)_6$ was used to host the working, counter, and Ag/AgCl reference electrodes in an electrochemical mini cell. The potential was linearly scanned from -0.60 V to 0.60 V vs. Ag/AgCl at 100 mV/s unless otherwise mentioned, and the effect of target epitopes on cyclic voltammetry (CV) peak currents of the $Fe(CN)_6^{3-}/Fe(CN)_6^{4-}$ redox probe was examined [5,6]. In addition, ac impedance was characterized (ZENNIUM/IM6, Zahner-electrik GmbH& Co KG) for epitope-templated MIP and NIP film-coated electrodes [7]. Measurements were performed using a 1.0 pg/mL PA and the $Fe(CN)_6^{3-}/Fe(CN)_6^{4-}$ solution. The A549 (2×10^4) cells were seeded in 24-well cell plates and kept at 37 °C in 5 vol% CO_2 for 24 h. Then, the culture medium was collected and diluted on the third day for the MMP-1 determinations. The human MMP-1 enzyme-linked immunosorbent assay (ELISA) kit (#EHMMP1CL, Invitrogen - Thermo Fisher Scientific), which was purchased for determining the A549 culture medium, and the procedure used followed the product sheet (https://assets.thermofisher.com/TFS-Assets/LSG/manuals/EHMMP1CL.pdf).

S1.5 Immunohistochemistry of MMP-1 Protein

The A549 (2×10^4) cells were seeded in 24-well cell plates and kept at 37 °C in 5 vol% CO_2 for 24 h, and then the culture medium was collected for the MMP-1 determinations. Cells were then washed with 400 μL of the PBS (pH = 7.4) solution in each well and fixed in 3.7% formaldehyde in the PBS (pH = 7.4) solution for 10 min at room temperature. After that, each well was washed with 350 μL of the PBS (pH = 7.4) solution, then cells were permeabilized with 1% Triton X-100 for 5 min at room temperature, then washed again with 350 μL of the PBS (pH = 7.4) solution followed by blocking of nonspecific binding by washing in the PBS (pH = 7.4) solution supplemented with 5% bovine serum albumin (BSA) for 60 min. Finally, 350 μL of the PBS(pH = 7.4) solution was added to each well, and then cells were incubated overnight at 4 °C with the rabbit anti-(MMP-1) antibody (Sino Biological, #101282-T02) to BSA ratio of 1 : 250. Subsequently, the cells were trice washed in the PBS (pH = 7.4) solution for 5 min each time and labeled with 300 μL/well secondary antibody for 1 h at room temperature. After another washing in 250 μL of the PBS (pH = 7.4) solution at room temperature, the cells were co-stained with the 4',6-diamidino-2-phenylindole DAPI (Sigma) nuclear dye for 15 min. Finally, the cells were washed with the PBS (pH = 7.4) solution, then imaged with a CKX41 Olympus inverted fluorescence microscope (Melville, NY, USA).

Table S1. Peptides and their physico-chemical properties resulting from in silico cleavage of the MMP-1 biomarker.

Peptide	No. of Amino Acids	The Cutting Enzyme(s)	Location in Protein	Total Score	E value	*M*	pI	Instability Index	GRAVY	Aggregation "Hot Spots"
MIAHDFPGIGHK	12	Trypsin	Outside	43.5	1.E-06	1322.55	6.69	24.52	-0.058	0
TKVSEGQADIMIS	13	Pepsin pH = 1.3, Pepsin > 2.0	Partially hidden	43.9	1.E-06	1378.56	4.37	10.98	-0.015	0
TYRIENYTPDL	11	Pepsin pH = 1.3	Hidden	40.0	8.E-06	1384.51	4.37	19.09	-1.118	0
PRADVDHAIEKA	12	Pepsin pH = 1.3, Pepsin > 2.0	Outside	40.9	1.E-05	1321.46	5.39	19.90	-0.800	0
FQPGPGIGGDAH	12	Pepsin pH = 1.3, Pepsin > 2.0	Hidden	40.5	1.E-05	1152.23	5.08	21.35	-0.492	0
AQDDIDGIQAI	11	Pepsin pH > 2.0	Outside	38.0	1.E-04	1158.23	3.42	18.88	-0.073	0
HGYPKDIYSS	10	Pepsin pH = 1.3	Outside	36.3	3.E-04	1166.26	6.74	21.74	-1.230	0
WEQTHLTYR	9	Trypsin	Hidden	35.8	4.E-04	1233.35	6.75	11.50	-1.611	0
SEENTGKTYF	10	Pepsin pH = 1.3	Hidden	35.4	6.E-04	1175.22	4.53	35.39	-1.550	0
ADVDHAIEK	9	Trypsin	Outside	31.6	0.01	997.07	4.54	31.58	-0.589	0
HGYPKDIY	8	Pepsin pH > 2.0	Outside	31.2	0.013	992.10	6.74	0.60	-1.337	0

SMDPGYPK	8	Trypsin	Outside	30.3	0.027	894.01	5.55	13.65	-1.400	0
WAVQGQNV	8	Pepsin pH > 2.0	Outside	29.9	0.038	900.99	5.52	-10.04	-0.200	0
ADRDEVRF	8	Pepsin pH = 1.3, Pepsin > 2.0	Hidden	29.5	0.053	1007.07	4.56	-11.29	-1.337	0
SHSTDIGA	8	Pepsin pH = 1.3, Pepsin pH > 2.0	Outside	26.9	0.43	786.80	5.06	-1.86	-0.388	0
HGTRQYK	7	Pepsin pH = 1.3	Hidden	26.5	0.49	888.98	9.99	-1.53	-2.500	0
HRVAAHE	7	Pepsin pH = 1.3, Pepsin pH > 2.0	Hidden	25.7	0.98	818.89	6.92	-3.56	-0.943	0
FDGPGGN	7	Pepsin pH = 1.3, Pepsin pH >2.0	Outside	24.8	2.00	662.66	3.80	31.70	-1.000	0
DAITTIRGEVM	11	Pepsin pH = 1.3, Pepsin pH > 2.0	Hidden	38.8	5.E-05	1205.39	4.37	-5.48	0.327	0
LTFDAITTIR	10	Trypsin	Hidden	35.0	9.00E-04	1150.34	5.84	33.68	0.730	0
DGFFYFFHGTR	11	Trypsin	Hidden	41.4	6.E-06	1393.52	6.74	21.58	-0.255	1
DIYSSFGFPR	10	Trypsin	Outside	36.3	3.E-04	1188.30	5.84	39.98	-0.280	1
TYFFVANK	8	Trypsin	Hidden	29.9	0.038	989.14	8.26	38.35	0.275	1

GEVMFFK	7	Trypsin	Hidden	27.4	0.24	857.04	6.00	-23.67	0.557	1
TEGNPRWEQTH	11	Pepsin pH = 1.3	Partially hidden	41.4	6.E-06	1354.40	5.37	78.99	-2.364	0
IENYTPDLPR	10	Trypsin	Outside	37.1	2.E-04	1217.14	4.37	48.63	-1.190	0
DEDERWTNN	9	Pepsin pH = 1.3	Hidden	35.4	5.E-04	1178.14	3.92	60.53	-3.011	0
VRGDHRDNSP	10	Pepsin pH = 1.3, Pepsin pH > 2.0	Outside	35.4	6.E-04	1152.19	6.72	44.45	-2.130	0
FDPKTKRILT	10	Pepsin pH = 1.3, Pepsin pH > 2.0	Outside	35.4	6.E-04	1218.46	9.99	73.20	-0.770	0
PRTVKHIDAA	10	Pepsin pH = 1.3, Pepsin pH > 2.0	Hidden	34.6	0.001	1107.28	9.18	42.52	-0.510	0
KRSMDPGY	8	Pepsin pH > 2.0	Outside	30.3	0.027	953.08	8.59	93.81	-1.762	0

M – molecular weight; pI – Isoelectric point.

Table S2. The comparison of sensing methods for MMP-1 (modified and added from [8]).

Sensing element	Substrate	Detection method	Sensing range	Limit of detection	Ref.
Antibody	Au Chip	SPRI	0.05-20.00 ng/mL (0.93 pM– 370 nM)	9 pg/mL (0.17 pM)	[9]
Particular MMP substrates	Au NP/MCH-gelatin/ casein/collagen	Colorimetry	10-700 ng/mL (0.19-13.0 nM)		[10]
A fluorogenic MMP peptide substrate	pSiRM	Fluorescence	1 pM– 100 nM	0.75 aM	[11]
MIPs	Au/Ti	EG-FET	50-500 nM		[12]
pAIPs	ITO	CV	0.001 – 10.0 pg/mL (0.02 fM – 0.19 pM)	0.2 fg/mL (3.7 aM)	This work

Abbreviations: Au NPs: gold nanoparticles; SPRI: surface plasmon resonance imaging; MMP: matrix metalloproteinase; MCH: 6-mercaptohexan-1-ol; pSiRM: porous silicon resonant microcavity; MIPs: molecularly imprinted polymers. EG-FET: extended-gate field-effect transistor; pAIP: peptide A-imprinted polymer; ITO: indium-tin-oxide; CV: cyclic voltammetry.

Table S3. MMP-1 determination in the culture medium of A549 cells using the MIPs film-coated electrode chemosensors and the ELISA. The standard deviations are based on at least three individual measurements.

Sample No.	MIP (μA/cm²)	Converted conc. (ng/mL)	Average MMP-1 conc. (ng/mL)		Accuracy (%)
			pAIPs	ELISA	
1	552	752.5			
2	554	883.3	796.1± 61.7	784.0± 33.9	101.5
3	552	752.5			

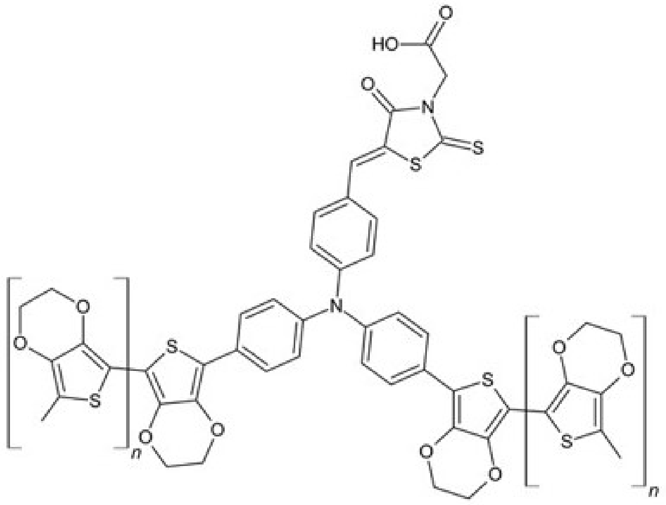

Scheme S1. A proposed structural formula of the pA-imprinted poly(TPARA-*co*-EDOT).

```
>sp|P03956|MMP1_HUMAN Interstitial collagenase OS=Homo sapiens OX=9606 GN=MMP1 PE=1 SV=3
MHSFPPLLLLLFWGVVSHSFPATLETQEQDVDLVQKYLEKYYNLKNDGRQVEKRRNSGPV
VEKLKQMQEFFGLKVTGKPDAETLKVMKQPRCGVPDVAQFVLTEGNPRWEQTHLTYRIEN
YTPDLPRADVDHAIEKAFQLWSNVTPLTFTKVSEGQADIMISFVRGDHRDNSPFDGPGGN
LAHAFQPGPGIGGDAHFDEDERWTNNFREYNLHRVAAHELGHSLGLSHSTDIGALMYPSY
TFSGDVQLAQDDIDGIQAIYGRSQNPVQPIGPQTPKACDSKLTFDAITTIRGEVMFFKDR
FYMRTNPFYPEVELNFISVFWPQLPNGLEAAYEFADRDEVRFFKGNKYWAVQGQNVLHGY
PKDIYSSFGFPRTVKHIDAALSEENTGKTYFFVANKYWRYDEYKRSMDPGYPKMIAHDFP
GIGHKVDAVFMKDGFFYFFHGTRQYKFDPKTKRILTLQKANSWFNCRKN
```

Figure S1. FASTA form of matrix metalloproteinase-1 (MMP-1) biomarker.

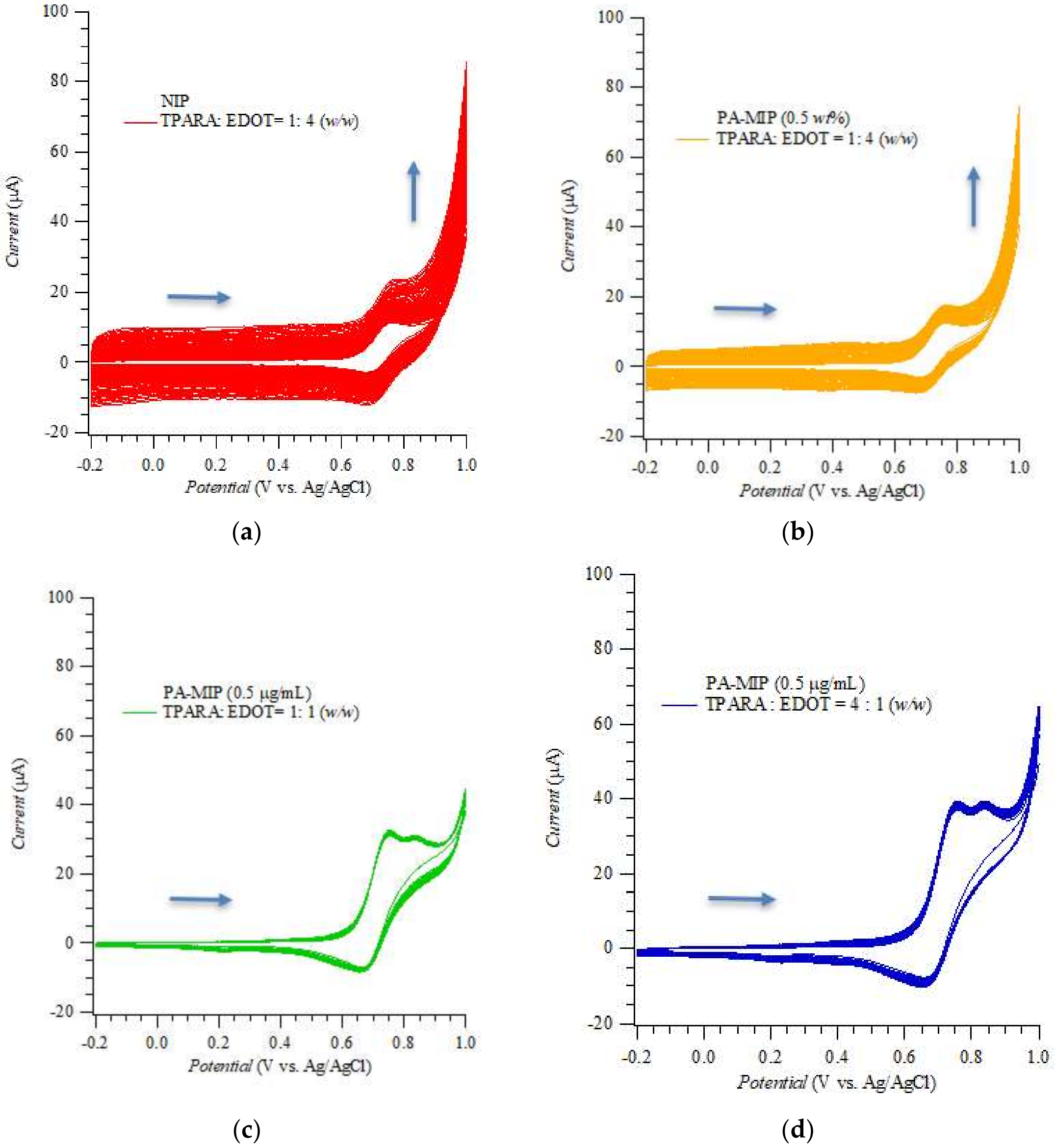

Figure S2. Potentiodynamic curves for deposition of (**a**) non-imprinted (NIP) and peptide molecularly imprinted polymer (MIP) films with 0.50 μg/mL PA present during the electropolymerization in a TPARA : EDOT (**b**) 1 : 4 (*wt*/*wt*), (c) 1 : 1, and (d) 4 : 1 solution on a 0.5 × 0.5 cm² area ITO electrodes.

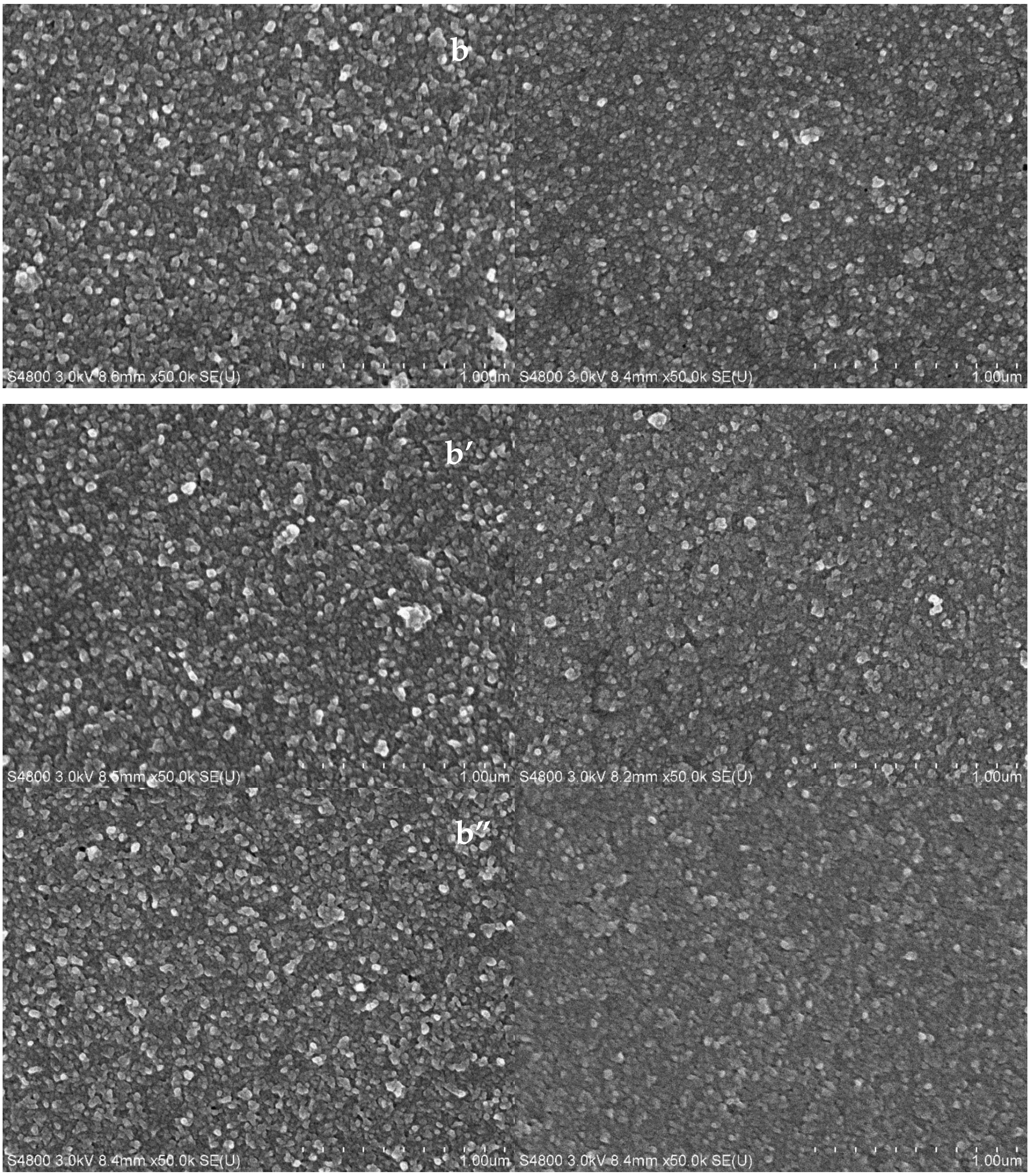

Figure S3. SEM images of (**a**) NIPs, and (**b**) PA-templated poly(TPARA-*co*-EDOT) film-coated electrodes (**a**') before, (**b**') after PA template removal with ethanol, and (a'' and b'') after PA analyte binding from the 1 pg/mL PA solution for 30 min.

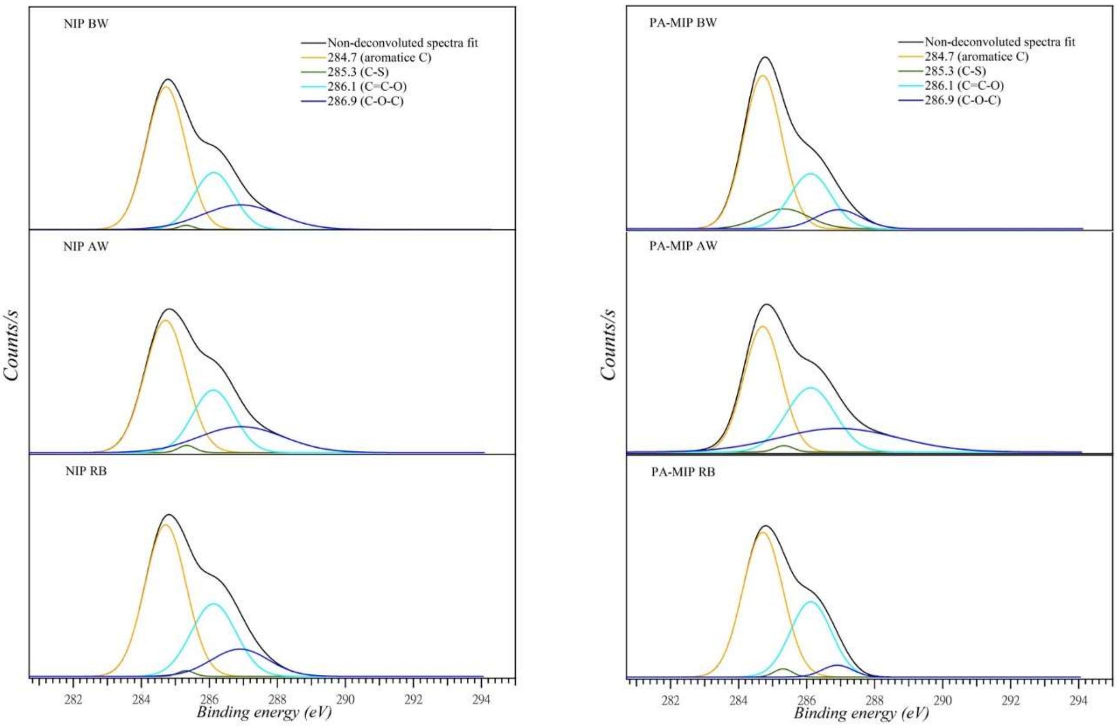

(a) **(b)**

Figure S4. The XPS spectra of non-deconvoluted and deconvoluted C 1s bands for the (**a**) NIP and (**b**) PA-templated poly(TPARA-*co*-EDOT) (PA-MIP) films. BW – before washing; AW – after washing; RB – after PA analyte binding from the 1.0 pg/mL PA solution for 30 min.

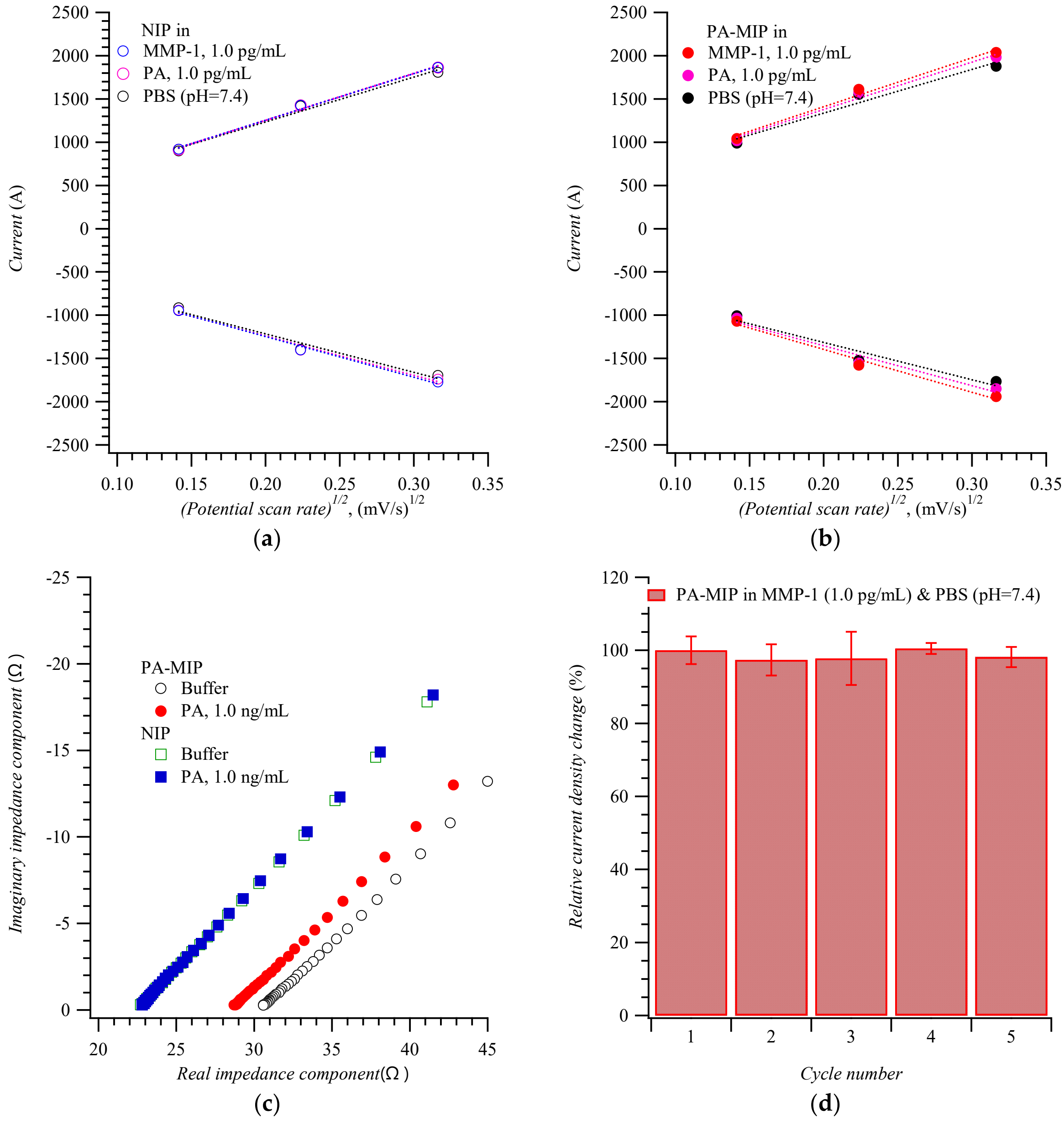

Figure S5. Curves of the CV peak dependence on the square root of a potential scan rate for (**a**) the NIP and (**b**) MIP Figure 5. mM $K_4Fe(CN)_6$ and 5 mM $K_3Fe(CN)_6$, 1.0 pg/mL PA and 1.0 pg/mL MMP-1, in the PBS (pH = 7.4) solution, fitted with the Randles-Ševčik equation. (**c**) The ac impedance imaginary vs. real component dependence for the MIP and NIP film-coated electrodes in 5 mM $K_4Fe(CN)_6$ and 5 mM $K_3Fe(CN)_6$, in the 1.0 pg/mL pA, PBS (pH = 7.4) solution and a similar solution without PA. (**d**) The PA-templated (pAIPs) poly(TPARA-co-EDOT) film-coated electrodes reusability. The electrode was used to determine MMP-1 in its 1.0 ng/mL solution, rinsed, then consecutively reused five times.

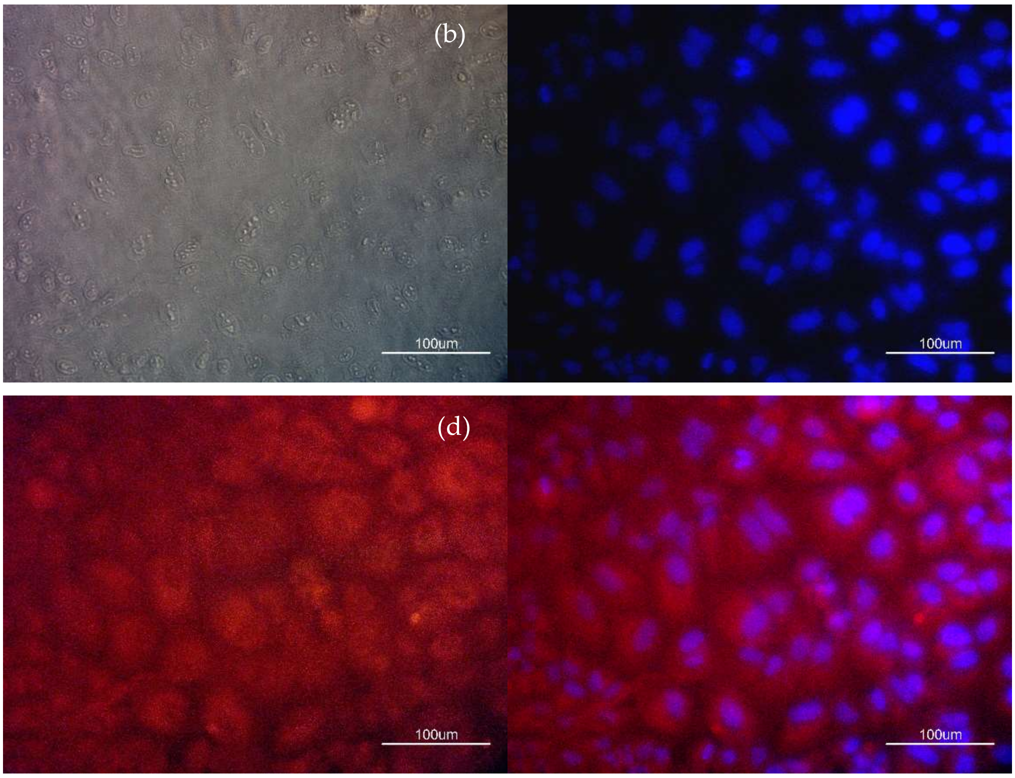

Figure S6. (**a**) Optical, (**b**) DAPI staining, (**c**) immunohistochemistry of MMP-1 proteins, and (**d**) merge images of A549 lung cancer cells at 400× magnification.

References

1. Yang, C.-H.; Huang, L.-R.; Chih, Y.-K.; Lin, W.-C.; Liu, F.-J.; Wang, T.-L. Molecular assembled self-doped polyaniline copolymer ultra-thin films. *Polymer* **2007**, *48*, 3237–3247, http://dx.doi.org/10.1016/j.polymer.2007.04.013.
2. Behl, M.; Hattemer, E.; Brehmer, M.; Zentel, R. Tailored Semiconducting Polymers: Living Radical Polymerization and NLO-Functionalization of Triphenylamines. *Macromol. Chem. Phys.* **2002**, *203*, 503–510, https://doi.org/10.1002/1521-3935(20020201)203:3<503::AID-MACP503>3.0.CO;2-P.
3. Yang, C.-H.; Chen, H.-L.; Chuang, Y.-Y.; Wu, C.-G.; Chen, C.-P.; Liao, S.-H.; Wang, T.-L. Characteristics of triphenylamine-based dyes with multiple acceptors in application of dye-sensitized solar cells. *J. Power Sources* **2009**, *188*, 627–634, https://doi.org/10.1016/j.jpowsour.2008.12.026.
4. Lee, M.-H.; O'Hare, D.; Guo, H.-Z.; Yang, C.-H.; Lin, H.-Y. Electrochemical sensing of urinary progesterone with molecularly imprinted poly(aniline-co-metanilic acid)s. *J. Mater. Chem. B* **2016**, *4*, 3782–3787, https://doi.org/10.1039/C6TB00760K.
5. Huang, C.-Y.; Tsai, T.-C.; Thomas, J.L.; Lee, M.-H.; Liu, B.-D.; Lin, H.-Y. Urinalysis with molecularly imprinted poly(ethylene-co-vinyl alcohol) potentiostat sensors. *Biosens. Bioelectron.* **2009**, *24*, 2611–2617 https://doi.org/10.1016/j.bios.2009.01.016.
6. Huang, C.-Y.; O'Hare, D.; Chao, I.J.; Wei, H.-W.; Liang, Y.-F.; Liu, B.-D.; Lee, M.-H.; Lin, H.-Y. Integrated potentiostat for electrochemical sensing of urinary 3-hydroxyanthranilic acid with molecularly imprinted poly(ethylene-co-vinyl alcohol). *Biosens. Bioelectron.* **2015**, *67*, 208–213, http://dx.doi.org/10.1016/j.bios.2014.08.018.
7. Lee, M.-H.; Thomas, J.L.; Su, Z.-L.; Zhang, Z.-X.; Lin, C.-Y.; Huang, Y.-S.; Yang, C.-H.; Lin, H.-Y. Doping of transition metal dichalcogenides in molecularly imprinted conductive polymers for the ultrasensitive determination of 17β-estradiol in eel serum. *Biosens. Bioelectron.* **2020**, *150*, 111901, https://doi.org/10.1016/j.bios.2019.111901.
8. Lei, Z.; Jian, M.; Li, X.; Wei, J.; Meng, X.; Wang, Z. Biosensors and bioassays for determination of matrix metalloproteinases:

state of the art and recent advances. *J. Mater. Chem. B* **2020**, *8*, 3261–3291, https://doi.org/10.1039/C9TB02189B.
9. Tokarzewicz, A.; Romanowicz, L.; Sveklo, I.; Gorodkiewicz, E. The development of a matrix metalloproteinase-1 biosensor based on the surface plasmon resonance imaging technique. *Analytical Methods* **2016**, *8*, 6428-6435, https://doi.org/10.1039/C6AY01856D.
10. Chuang, Y.-C.; Huang, W.-T.; Chiang, P.-H.; Tang, M.-C.; Lin, C.-S. Aqueous zymography screening of matrix metalloproteinase activity and inhibition based on colorimetric gold nanoparticles. *Biosens. Bioelectron.* **2012**, *32*, 24–31, https://doi.org/10.1016/j.bios.2011.11.002.
11. Krismastuti, F.S.H.; Pace, S.; Voelcker, N.H. Porous Silicon Resonant Microcavity Biosensor for Matrix Metalloproteinase Detection. *Adv. Funct. Mater.* **2014**, *24*, 3639–3650, https://doi.org/10.1002/adfm.201304053.
12. Bartold, K.; Iskierko, Z.; Borowicz, P.; Noworyta, K.; Lin, C.-Y.; Kalecki, J.; Sharma, P.S.; Lin, H.-Y.; Kutner, W. Molecularly imprinted polymer-based extended-gate field-effect transistor (EG-FET) chemosensor for selective determination of matrix metalloproteinase-1 (MMP-1) protein. *Biosens. Bioelectron.* **2022**, *208*, 114203, https://doi.org/10.1016/j.bios.2022.114203.
